# An open-source molecular builder and free energy preparation workflow

**DOI:** 10.1038/s42004-022-00754-9

**Published:** 2022-10-27

**Authors:** Mateusz K. Bieniek, Ben Cree, Rachael Pirie, Joshua T. Horton, Natalie J. Tatum, Daniel J. Cole

**Affiliations:** 1 School of Natural and Environmental Sciences, Newcastle University, Newcastle upon Tyne, NE1 7RU UK; 2 Newcastle University Centre for Cancer, Translational and Clinical Research Institute, Newcastle University, Newcastle upon Tyne, NE2 4HH UK

**Keywords:** Computational chemistry, Computational chemistry, Structure-based drug design

## Abstract

Automated free energy calculations for the prediction of binding free energies of congeneric series of ligands to a protein target are growing in popularity, but building reliable initial binding poses for the ligands is challenging. Here, we introduce the open-source FEgrow workflow for building user-defined congeneric series of ligands in protein binding pockets for input to free energy calculations. For a given ligand core and receptor structure, FEgrow enumerates and optimises the bioactive conformations of the grown functional group(s), making use of hybrid machine learning/molecular mechanics potential energy functions where possible. Low energy structures are optionally scored using the gnina convolutional neural network scoring function, and output for more rigorous protein–ligand binding free energy predictions. We illustrate use of the workflow by building and scoring binding poses for ten congeneric series of ligands bound to targets from a standard, high quality dataset of protein–ligand complexes. Furthermore, we build a set of 13 inhibitors of the SARS-CoV-2 main protease from the literature, and use free energy calculations to retrospectively compute their relative binding free energies. FEgrow is freely available at https://github.com/cole-group/FEgrow, along with a tutorial.

## Abstract (web summary)

Automated free energy calculations for the prediction of binding free energies of ligands to a protein target are gaining importance for drug discovery, but building reliable initial binding poses for the ligands is challenging. Here, the authors introduce an open-source workflow for building user-defined congeneric series of ligands in protein binding pockets for input to free energy calculations.

## Introduction

Computational structure-based molecular design, in particular aiding the discovery of novel chemicals with desired biological activity, plays a crucial role in the modern drug discovery pipeline. High-throughput virtual screening is widely used in hit discovery^1^, but relies on pre-defined libraries of compounds. De novo design software packages aim to construct a model of a ligand in a target binding pocket using growth algorithms, either starting from a scaffold of a known hit compound or entirely from scratch. Such approaches can be beneficial as they do not rely on a (physical or virtual) library, and molecules can be tailored specifically to the problem at hand. Advances in de novo design software have been extensively reviewed^2^, and examples include both rule-based generation methods such as OpenGrowth^3^, AutoGrow^4^, and LigBuilder^5^, and recently deep generative methods for molecule design^6^.

With advances like these described above, much progress has been made in the important problem of optimising a molecular design within the context of a pre-defined scoring function and binding pocket. However, whether the designed molecule indeed has high biological activity is crucially reliant on the accuracy of the methods that are used to generate and score poses of the designed molecules, as well as other assumptions, such as a rigid receptor, that might be employed. Furthermore, the generated molecules can be quite esoteric, which may be advantageous with regards to arriving at novel intellectual property, but may not be ideal from a synthetic tractability viewpoint^7^. More commonly, a drug discovery effort may have identified a hit compound with a well-defined binding mode and wish to explore structure-activity relationships amongst a small library of synthetically accessible analogues. In this case, it would be beneficial to make use of prior knowledge about the binding mode when generating poses of designed compounds. One example of this approach is the E-novo workflow^8^, which was made available in Pipeline Pilot or Discovery Studio. The available conformations of added chemical functional groups (R-groups) were enumerated with a rigid core, and scored using a CHARMM-based docking method. The physics-based molecular mechanics-generalised Born with surface area (MM-GBSA) was then used to provide a more accurate score. Further, more recent, examples include FragExplorer^9^, which aims to grow or replace fragments to optimise molecular interaction fields generated by the GRID software^10^, DeepFrag^11^, which predicts appropriate fragment additions using a deep convolutional neural network trained on thousands of known protein–ligand complexes, and DEVELOP^12^, which uses deep generative models to output 3D molecules conditional on provided phamacophoric features of the binding site. However, the employed approximate physics- or knowledge-based approaches to scoring the designs will limit to some extent their ability to predict and optimise binding affinity.

On the other hand, free energy methods are much more computationally expensive approaches to molecular design that employ rigorous thermodynamics and carefully parameterised force fields to compute (relative or absolute) protein–ligand binding free energies. As such, they overcome many of the accuracy limitations of de novo design workflows, and are commonly employed in prospective design efforts to explore and prioritise relatively small perturbations in the hit-to-lead stage^13^. Many excellent tutorials and best practice documentation are available^14–18^, but most start from the assumption that the user has already built initial poses of the ligands in the binding pocket. For simple R-group additions, input coordinates may be built from maximum common substructure alignment, for example, but it may be difficult to resolve steric clashes or decide between two alternative 3D poses in more complicated cases^16^. Some widely-used graphical user interfaces, such as Maestro^19^ and Chimera^20^, are also available for building R-groups, but these can be proprietary and/or difficult to build into automated workflows and modify according to user needs.

Notable successful computer-aided design efforts have used free energy calculations in conjunction with de novo design tools to build (and maybe score) new molecules. Jorgensen and co-workers^13^ have pioneered this approach for many years, linking de novo design through the biochemical and organic model builder (BOMB) software with free energy perturbation (FEP) through the MCPRO software. BOMB builds ligands into a binding pocket by linking user-defined R-groups to an existing core. Functionality is available for conformer searching, structural optimisation, and scoring, using a custom scoring function trained via linear regression on > 300 experimental activity data points^21^. Once hits have been built and scored, hit-to-lead optimisation may be further refined through free energy calculations. Such an approach has yielded extremely potent series of leads against HIV reverse transcriptase^22^, macrophage migration inhibitory factor^23^, and the SARS-CoV-2 main protease^24^. In other drug discovery programmes, as part of the recent COVID Moonshot open science effort to crowd source design of SARS-CoV-2 main protease inhibitors^25^, the Omega toolkit by OpenEye^26^ is used for constrained conformer generation, and optimal binding poses are then taken through to free energy calculations using the perses software^27^. The evident importance of input structure to the reliability of free energy calculations^16^ means that open-source tools to automate this step are crucial.

Inspired by the BOMB/MCPRO approach to molecular design^13^, we introduce here the FEgrow open-source workflow for growing functional groups, chosen by the user, from a defined position on a core compound. To account for the multi-objective nature of molecular design, we output simple rule-of-five indicators of oral bioavailability, as well as flags for undesirable substructures and synthetic accessibility estimates. For the designed ligands, we enumerate 3D conformers of the added R-group, with options for additional flexibility if desired, within the context of the protein (discarding conformers with steric clashes). A common issue with generating docked poses is inaccuracy in the molecular mechanics force fields used to refine them, particularly for uncommon chemistries. To overcome this, we employ a hybrid machine learning/molecular mechanics (ML/MM) approach to optimisation, whereby the ligand is (optionally) described by the ANI neural network potential^28,29^, and non-bonded interactions with the static protein are described by traditional force fields. The binding affinities of low energy poses are predicted using a deep learning-based scoring function. Finally, FEgrow outputs binding poses in a form suitable for input to free energy calculations, and we illustrate this process with a case study, using the SOMD software^30^ to retrospectively compute relative binding free energies of several inhibitors of the SARS-CoV-2 main protease^24^.

In this way, we hope to integrate medicinal chemistry expertise in the FEgrow design workflow, with state-of-the-art methods for pose prediction, scoring, and free energy calculation. By building ligands from the constrained core of a known hit, we maximise the use of input from structural biology, and reduce reliance on docking algorithms. We aim for an open-source, customisable, fast, and easy-to-use (accessed through Jupyter notebooks) workflow that can adapt to community needs and advances in molecular design.

## Results

### Workflow design

**Fig. 1:**
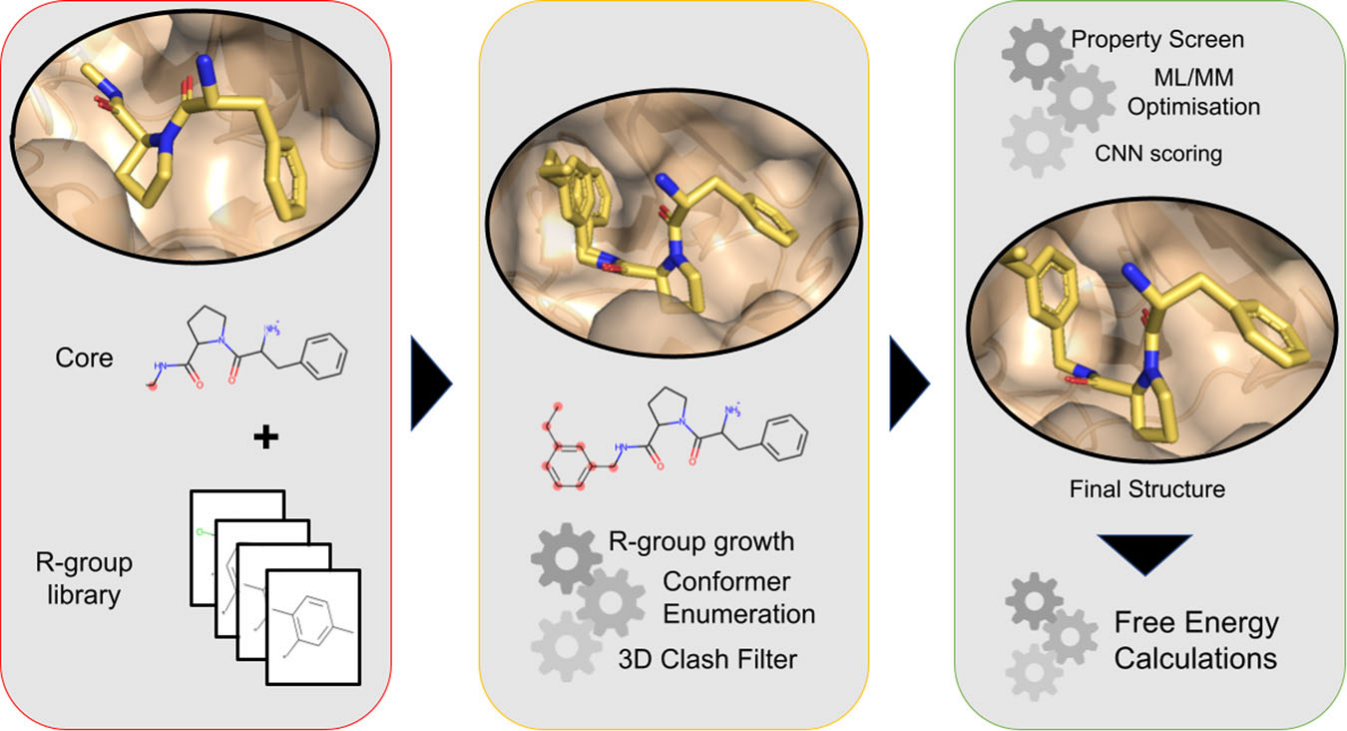
Overview of the FEgrow workflow. (left) The user specifies the receptor, ligand core, and a list of functional groups, along with their attachment points. (centre) RDKit^32^ is used to attach the selected R-group(s) and enumerate the available conformers with a rigid core. (right) Possible bioactive conformers undergo structural optimisation using a hybrid ML/MM potential energy function. The binding affinity is predicted using a convolutional neural network scoring function^47^ and molecular properties are assessed. Final structures are output for further free energy based binding affinity assessment.

The FEgrow package is written in Python, and supports Jupyter visualisation at each stage using py3Dmol^31^. Underneath, the main unit in the package is RMol which extends the RDKit class rdkit.Chem.rdchem.Mol^32^ with additional functionalities, such as visualisation, molecule merging, conformer generation, as well as storage of energies and other metadata. A convenience class RList is provided with the same functions for operating on a set of molecules, which allows also for future parallelisation. A modular workflow allows for addition/removal of functionality, such as new scoring functions or optimisation algorithms. FEgrow is freely available at https://github.com/cole-group/FEgrow, along with a tutorial. Figure 1 shows the overall design of the FEgrow workflow, and the component methods are described in the following sections.

#### Input and constrained conformer generation

The first task is to define the receptor and the ligand core, along with an attachment point for growth (currently only growth from hydrogen atoms is supported). Users may download receptor and ligand structures directly from the protein databank (PDB), or upload pre-prepared structures. In this study, we used the Open Babel software^33^ for parsing input structure files and ligand protonation (at pH 7).

Merging the ligand core with a new R-group requires that both the linking atom on the template core and on the attachable R-group are specified. The merging is carried out with the RDKit editable molecule^32^. RDKit is further used to generate 3D conformers using the ETKDG method^34^. The generated conformers are aligned, and energy minimised using the Universal Force Field^35^. Harmonic distance restraints to their initial positions are applied to atoms in the common core (identified by a maximum common structure search) using a stiff force constant (10^4^ kcal/mol/Å^2^). In this way, we can enforce the conformations of the generated molecules to only vary from the core in the region of the added R-group. This region may additionally be extended by adding further atoms into the flexible substructure of the template. For convenience, we provide a minimal set of around 500 R-groups that are commonly used in medicinal chemistry optimisation^36^. R-groups can be interactively selected from the library using the mols2grid package^37^, or the user may prepare their own molecules for attachment (see Tutorial).

#### Geometry optimisation

The constrained conformer generation described above aims to enumerate all accessible, physically-reasonable conformers of the added R-group (and any other flexible regions) in vacuum. However, most of these conformations will be incompatible with the protein binding site. Hence, a 3D filter and geometry optimiser aims to find the bioactive conformers of the designed ligands.

The protein is treated with PDBFixer^38^ to add any missing atoms, residues, and hydrogen atoms. Water molecules (and other non-protein residues) are stripped by default, but can be optionally retained as part of the receptor, for example, if they are thought to form an important part of the hydrogen bonding network within the binding pocket (an example is shown later in Case Study I). A simple distance filter removes any ligand conformers that form a steric clash with the protein (any atom–atom distance less than 1 Å). Next, the remaining conformers are refined in the context of a rigid receptor via energy minimisation using OpenMM^38^. All atoms of the protein, and any retained water molecules, are kept fixed during the optimisation in the positions provided by the user.

The energy minimisation uses the AMBER FF14SB^39^ force field for the receptor and either GAFF2^40^ (General AMBER force field) or the Open Force Field 1.0.0 (‘Parsley’)^41^ general force fields for the ligand, with the choice left to the user. Optionally the intramolecular interactions of the ligand can be modelled using the ANI-2x ML potential^28^ in a hybrid ML/MM simulation. In this so-called mechanical embedding scheme, the total potential energy of the ML/MM system is composed of three terms^42^:

1 $${E}^{{{{{{{{\rm{tot}}}}}}}}}={E}^{{{{{{{{\rm{MM}}}}}}}}}(R)+{E}^{{{{{{{{\rm{MM}}}}}}}}}(RL)+{E}^{{{{{{{{\rm{ML}}}}}}}}}(L),$$

where R, RL, and L correspond to receptor–receptor, receptor–ligand, and ligand intramolecular interactions, respectively. The second term acts as the coupling term between the ML and MM subsystems and consists of the standard Coulomb and Lennard-Jones 12-6 non-bonded interaction energies. Thus, a general force field (here, GAFF2 or Parsley) is still required for the ligand to model the non-bonded interactions with the receptor. The use of ANI helps to avoid known deficiencies in the potential energy surfaces predicted by force fields, while ensuring that the optimisations are significantly faster than could be achieved with full quantum mechanics. For example, it has been shown that the description of biaryl torsions, which are commonly found in drug-like molecules, is one area where ANI-2x performs better than contemporary general force fields^43^. The hybrid ML/MM approach has also been shown to predict binding poses that overlap well with crystallographic electron density maps of bound ligands, even for those that contain charged moieties that were not included in the training of the ANI potential^44^. As such, in FEgrow, users may turn on the hybrid approach for binding pose refinement provided the molecule contains only elements covered by the model (H, C, N, O, F, S, and Cl), else the selected classical force field is used for the entire ligand.

Following the procedure recommended in BOMB, Lennard-Jones radii are scaled by a factor of 0.8 during optimisation. This is intended to mitigate to some extent the rigid protein approximation, by allowing extra space in the binding pocket to accommodate ligand growth. Furthermore, to account in an implicit manner for the neglected dielectric response of the protein and solvent molecules, the atomic charges are reduced by a factor of $\frac{1}{\sqrt{\epsilon }}$, where *ϵ* is the relative permittivity, in this case taken to be 4. Analysis of the effect of these scaling factors on structural and affinity predictions in Case Study I is provided in Supplementary Note 1, Table S1, and Fig. S1. The lowest energy optimised conformer, and all conformers within 5 kcal/mol, are output as PDB/SDF files for further analysis and scoring.

#### Binding pose scoring

Once the geometry optimisation is completed, the remaining (low energy) conformers are scored to predict their binding affinity. There are many choices available for scoring binding poses and their corresponding binding affinities, and these are usually classified as either force-field, empirical, or knowledge-based. In the latter case, input features (such as atom-atom pairwise contacts) are used to train models to reproduce data for known protein–ligand complexes. Recently, machine learning models have emerged, in which an arbitrary, nonlinear relationship between input and target prediction is learned. One such approach is the gnina convolutional neural network (CNN) model^45^, which takes as its input features a 3D grid-based representation of the protein–ligand complex and the atom types. The model has been jointly trained for binding pose and affinity prediction on a cross-docked set containing examples of ligand poses generated by docking into multiple receptors^46^. The resulting models are competitive with other grid-based CNN models, and outperform the traditional empirical Vina scoring function^46^. They are available as part of the gnina docking software package^47^, which is a fork of Smina^48^ and AutoDock Vina^49^. Here, we use gnina only for re-scoring the output ligand 3D structures, using the ‘score_only’ flag and the default ensemble of CNN scoring models. Gnina CNNaffinity scores (predicted pK) are output, and compared with experimental binding affinity (where available).

#### Molecular property filters

Having assembled the 2D and 3D structures of the core and user-defined R-groups, we include some simple tools for assessing the drug-likeness and synthetic tractability of the designed compounds. Several sets of rules exist to investigate the likelihood of a molecule displaying drug-like behaviour. While there are many examples of approved drugs which violate these considerations, they still provide a useful indication of whether a molecule is worth testing (that is, if it disobeys all of the conditions discussed below, it is most likely a poor candidate). FEgrow reports Lipinski’s rule of five (Ro5) counts,^50^ the synthetic accessibility score (SAScore)^51^ and flags describing whether the proposed molecule is Ro5 compliant and if it contains undesirable features based on the PAINS,^52^ NIH^53,54^ and unwanted substructure^55^ filters. Our implementation is adapted from the TeachOpenCADD^56^ Talktorials 2 and 3, using functionality from the Descriptors and FilterCatalog modules of RDKit.^32^ Further details are provided in Supplementary Note 2.

### Case study I: Protein–ligand benchmarks

The protein–ligand benchmark of Hahn et al.^57,58^ is an open, curated set of high quality structural (e.g., high similarity between crystallised and simulated ligands and no missing atoms) and bioactivity (e.g., taken from a single data source with adequate dynamic range) data, which has been collected with the goal of assessing the accuracy of free energy methods. For each target, modelled structures of the protein in complex with a congeneric series of ligands are provided as starting points for free energy calculations, but the methods used to position the R-groups are, to our knowledge, not necessarily consistent or documented.

**Fig. 2:**
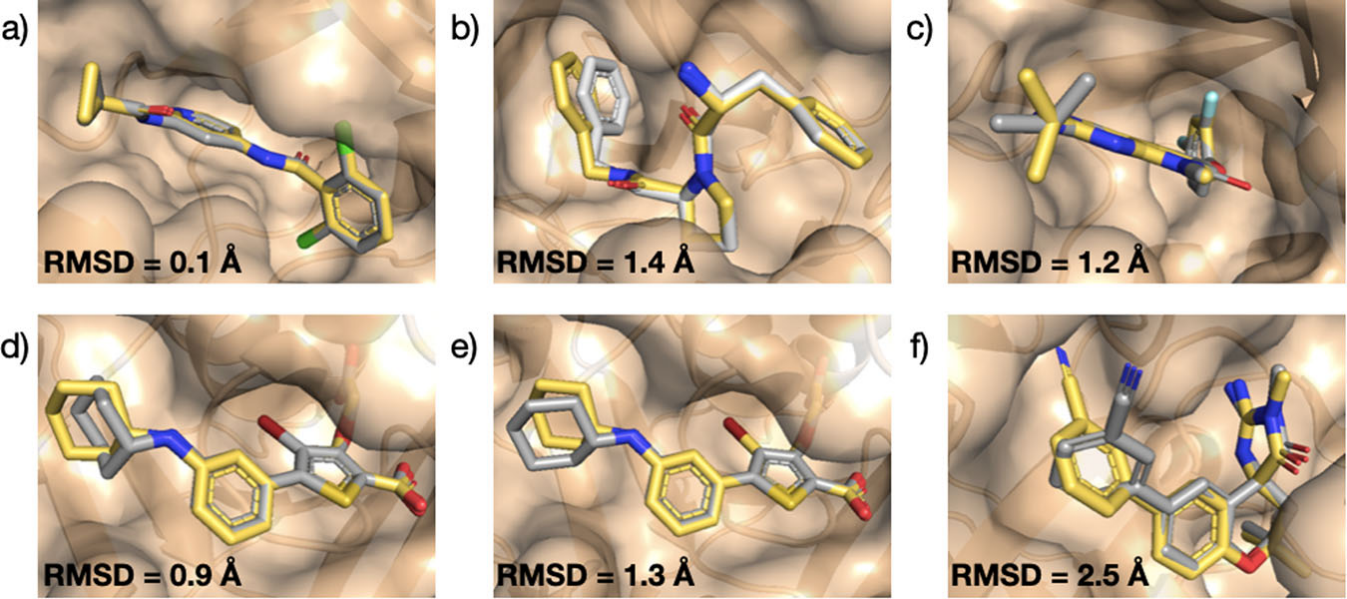
Overlay of experimental and predicted protein-ligand benchmark dataset structures. Crystal structures are shown in yellow and grown compounds in grey. **a** TYK2 (PDB: 4GIH^59^), **b** Thrombin (PDB: 2ZFF^60^), **c** P38 (PDB: 3FLY^61^), **d** PTP1B with force field optimisation (PDB: 2QBS^62^), **e** PTP1B using ML/MM optimisation, and **f** BACE(Hunt) (PDB: 4JPC^63^). Root-mean-square distances (RMSD) between predicted and experimental coordinates of atoms in the built R-groups were calculated using RDKit^32^.

Here, we apply the FEgrow workflow to ten targets from the protein-ligand benchmark set. Starting from the crystal structure of each target, we truncate the bound ligand to a common core, which is shared across the congeneric series to be modelled. A summary of the targets, the crystal structures used, the number of R-groups grown, and their common core and net charge is provided in Tables S2 and S3. We use the methods outlined in the previous section to re-grow the congeneric series of ligands in the binding pockets, including enumeration and optimisation of possible R-group conformers, and scoring of final poses. Figure 2 shows overlays of the modelled and crystal structures (where there is an exact match between the crystallised ligand and one of the modelled R-groups), as well as the measured root-mean-square deviation (RMSD) between the predicted and experimental coordinates of the heavy atoms of the functional groups.

For the targets, TYK2 (Fig. 2a) and Thrombin (Fig. 2b), we obtain a good overlap between the grown R-groups and the crystal structures. In the former case, the dihedral angle formed between the grown cyclopropyl C2 and C3 carbons and the amide carbonyl oxygen core (30^∘^ and −37^∘^) are in agreement with those reported experimentally^59^. For Thrombin, although the added R-group here is a rigid phenyl moiety, we make use of the option to add atoms from the core to the flexible region and allow the linking –CH_2_– group to freely rotate during structural optimisation. This added flexibility leads to a rotation of the phenyl group of less than 10^∘^, compared to the corresponding crystal structure^60^.

The P38 benchmark set includes a series of alkyl amino substitutions originally investigated as part of a structure-activity relationship study into kinase inhibitors^61^. Here, the added amino group is correctly positioned to form a hydrogen bond with the protein backbone, though the *i*-Pr group is rotated by around 60° compared to the crystal structure (Fig. 2c). In PTP1B, the grown cyclohexyl substituent is able to rotate quite freely, with many conformations predicted to lie within 5 kcal/mol of the minimum. The minimum energy structure shows good overlap with the crystal structure^62^ with low RMSD, but connects to the core at the axial position of the cyclohexyl group (Fig. 2d). In this case, the core structure contains a Br atom, so we are unable to optimise with the ANI-2x potential (the workflow defaults to the Parsley force field for the ligand). Interestingly, if we remove the Br atom, and re-run the workflow using hybrid ML/MM optimisation, we recover the equatorial connection as the lowest energy conformer, in agreement with the crystal structure (Fig. 2e). This demonstrates the potential advantages of employing hybrid ML/MM structure prediction methods in binding mode determination.

Finally, the BACE(Hunt) target, includes a series of substituted phenyl additions to a spirocyclic core. Here, the grown cyanophenyl group is rotated by approximately 90°, relative to the crystal structure^63^, which shows the *meta*-CN group accommodated in the binding pocket (Fig. 2f). An exact match with the crystal structure is also output, but it is predicted to be around 3 kcal/mol higher in energy. Closer examination of the experimental structure reveals a crystal water molecule, close to the binding pocket, that is capable of forming a hydrogen bond with the –CN group, and a further network of water molecules that would be displaced by the conformation shown in Fig. 2f. Figure S2 investigates the effects of including the hydrogen-bonding water molecule in the rigid receptor structure, and changing the force fields used, but no input settings recover the crystal structure.

As discussed, we include with the FEgrow workflow the option to score the output poses of the designed ligands with a scoring function. In particular, we use the gnina convolutional neural network score, which has been trained on both binding pose and affinity prediction^46^. While accurate recovery of experimental binding affinity is not necessarily expected for current scoring functions, it is useful to evaluate to what extent they can be used to provide guidance in early stage design, ahead of more rigorous physics-based scoring methods. The root-mean-square error between gnina CNN affinities (converted to free energies) and experiment is quite acceptable (Table S4), ranging from 0.9 kcal/mol (BACE(P2)) to 1.7 kcal/mol (Jnk1), which indicates that the CNN scoring function is able to predict the affinity range of most of these series. In fact, the errors may be lower than typically expected^46^, because we are using here additional information from the experiment (the binding pose of the core) and not relying on the scoring function to determine the bioactive conformation.

The R^2^ correlation coefficients between the predicted and experimental affinities are more variable (Table S4), however, ranging from close to zero (the BACE targets) to 0.68 (Thrombin). The full set of CNN-predicted binding affinity data is plotted in Fig. S3, and reveals that most of the predictions lie in quite a narrow range, compared to the experimental data. We note that this is quite a challenging test for the scoring function, since the modifications made to the core are relatively small and cover a smaller dynamic range in affinity than most test sets. Nevertheless, it seems that current scoring functions have some utility in guiding design, but that more accurate physics-based scoring is required to accurately discriminate between structural changes in the hit-to-lead stage.

### Case study II: SARS-CoV-2 main protease

**Fig. 3:**
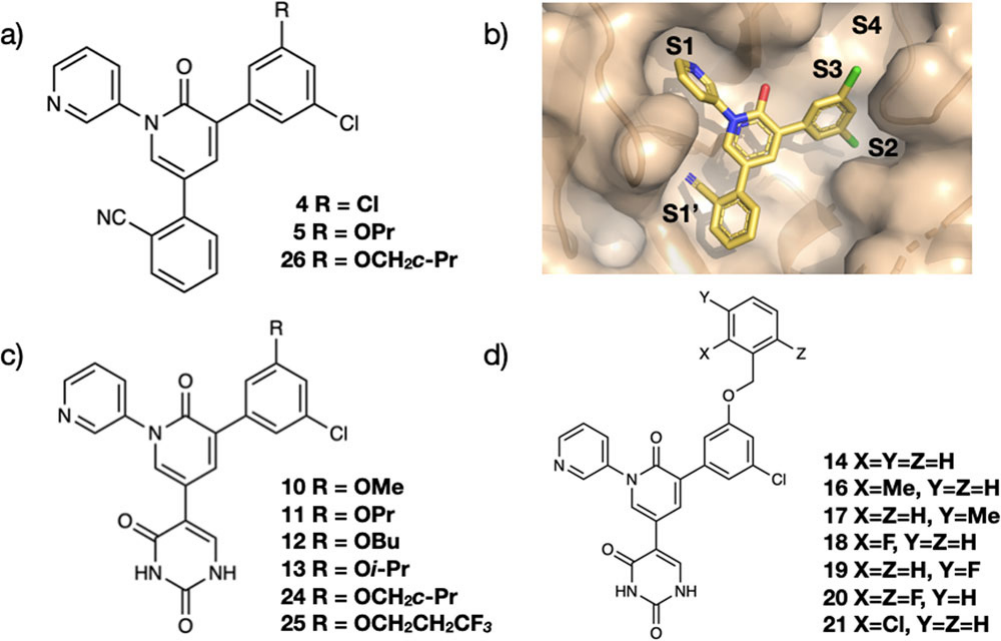
Structures of the series of cyanophenyl- and uracil-based compounds SARS-CoV-2 main protease (M^pro^) inhibitors investigated here. **a** Cyanophenyl-based M^pro^ inhibitors. **b** X-ray crystal structure of **4** in complex with the protease, with discussed binding pockets labelled. **c**, **d** Uracil-based M^pro^ inhibitors.

The main protease (M^pro^) of SARS-CoV-2, the virus responsible for the COVID-19 pandemic, is an attractive target for the development of antiviral agents^64^. The Jorgensen lab has focused on the development of drug-like, non-covalent inhibitors of the protease through lead optimisation of virtual screening hits^24^. In particular, starting from the anti-epileptic drug, perampanel, researchers combined model building with the BOMB software, with free energy calculations, to rapidly yield potent antiviral compounds. Figure 3 shows the structures of the two main series of cyanophenyl- and uracil-based compounds investigated. A high-resolution X-ray crystal structure of **4** with M^pro^ confirmed binding to the S1, S1’ and S2 pockets, with space to grow into the S3–S4 region^24^.

**Fig. 4:**
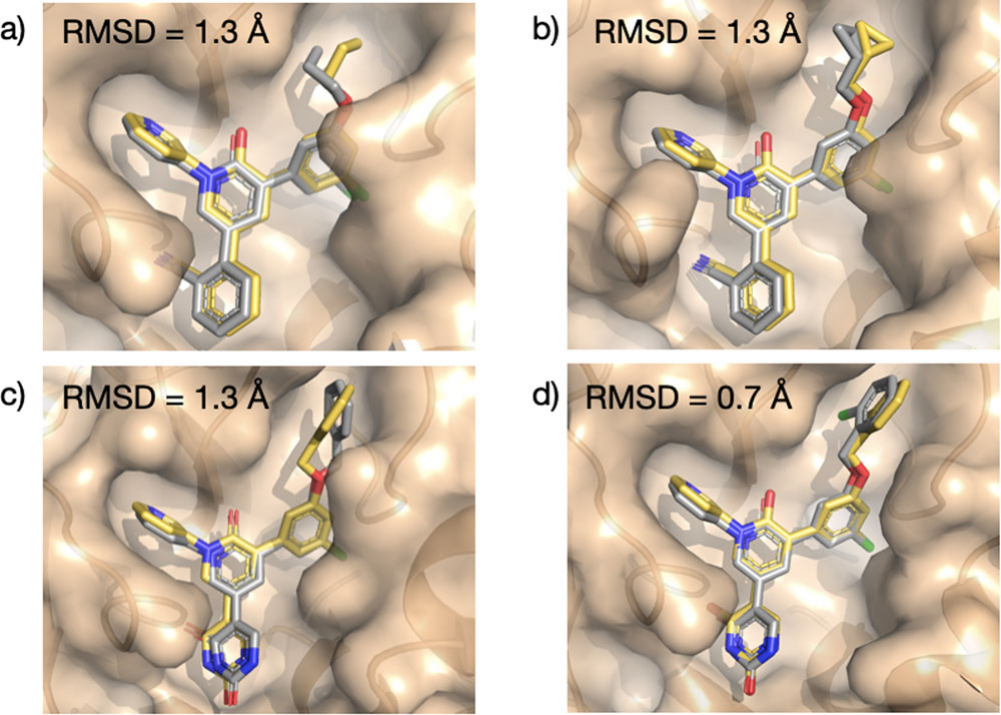
Comparison between experimental and predicted structures of SARS-CoV-2 main protease (M^pro^) inhibitors. Overlay of **a** **5** and PDBID: 7L11, **b** **26** and 7L14, **c** **14** and 7L12, **d** **21** and 7L13. Crystal structures are coloured in yellow, and modelled binding poses in grey. Root-mean-square distances (RMSD) between predicted and experimental coordinates of atoms in the built R-groups were calculated using RDKit^32^.

In what follows, we employ FEgrow to retrospectively build and score the listed analogues (Fig. 3) to demonstrate the potential utility of the workflow in guiding future design efforts. Starting from the crystal structure of **4** (PDBID: 7L10), we begin by replacing one of the *meta* chlorine atoms by propoxy to form **5**. The modelled structure agrees well with the corresponding high-resolution crystal structure (Fig. 4a). In particular, the propoxy OCCC dihedral angle in the lowest energy structure (53°) matches the experimental gauche conformation (47°), which allows hydrophobic contact with Met165 and Leu167. Similarly, good agreement is obtained for the cyclopropyl analogue **26** with the corresponding experimental crystal structure (Fig. 4b).

Turning attention to the uracil series, the core molecule was again built from the crystal structure of **4**, by removing the cyanophenyl group. The added uracil group has three low energy conformations, and in this case, we retained the second lowest energy structure, which forms key hydrogen bonding interactions with the backbone of Thr26 and the catalytic Cys145. In agreement with the original modelling, performed using the BOMB software^24^, we find that again a range of substituents are permitted in the S3/S4 pocket, including substituted benzyloxy side chains (Fig. 3). Figure 4c shows that the modelled uracil group in the S1’ pocket is in good agreement with the corresponding crystal structure (7L12). However, the predicted conformation of the unsubstituted benzyloxy side chain is at odds with the crystal structure (7L12). The correct conformer is output as an alternative low energy conformer and, interestingly, the majority of the modelled larger, substituted benzyloxy groups adopt the crystal conformation. This is exemplified by **21** in Fig. 4d, which also correctly orients the *ortho*-Cl down into the S4 pocket.

**Fig. 5:**
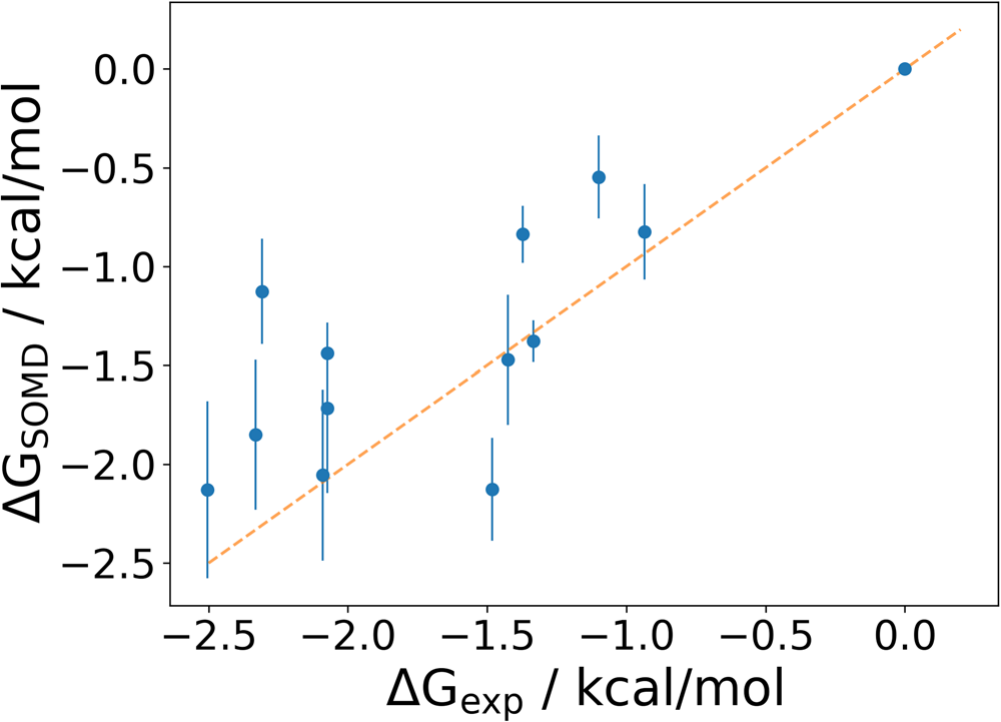
Comparison between free energy calculations and experiment. Binding free energies of 13 analogues of the uracil-based M^pro^ inhibitors, relative to compound **10**. The error bars indicate one standard error based on least square fitting^75^.

The uracil series comprises a set of 13 analogues, spanning around 2.5 kcal/mol in binding free energy, and as such provides a useful benchmark for demonstrating the next stage of the workflow. Although the gnina CNN affinities for these compounds are reasonably well correlated with experimental IC50 measurements in a kinetic assay (Fig. S5)^24^, it is desirable to investigate whether more rigorous free energy methods can be used to improve accuracy. Hence, relative binding free energies were computed using the SOMD software^30^, starting from the structures output by the FEgrow workflow in complex with the receptor (see “Computational Methods”). Note that we have used the lowest energy structures as input to the free energy calculations (using instead the structure of e.g., **14** that corresponds most closely to the crystal structure can introduce differences of up to 0.4 kcal/mol in free energies in our tests, but this information would not be available for prospective studies). Figure 5 shows the agreement between experiment and simulations (MUE = 0.45 kcal/mol, *R*^2^ = 0.53), and the raw data is provided in Table S5. Here, we can see that even though we have only used information from a single crystal structure of **4** bound to the protease, the combination of structure building and optimisation with the FEgrow workflow and free energy calculations with SOMD allows the (retrospective) prioritisation of compounds, such as compounds **20** and **21** for synthesis and testing.

## Discussion

We have introduced here FEgrow, an open-source molecular builder and free energy preparation workflow. Taking as input a receptor and ligand core structure, FEgrow aims to build a user-defined library of chemical functional groups of the sort that would typically be used to explore structure-activity relationships with free energy calculations. Inspired by the BOMB approach to molecular design^13^, we grow from a fixed ligand core in order to maximise the use of binding mode information from structural biology sources, and rely on the user’s medicinal chemistry expertise to suggest functional groups that improve binding affinity whilst remaining synthetically tractable. Alternative, generative methods for fragment growth^11,12^ could be incorporated in future, but testing of expert medicinal chemist designs still remains popular today and FEgrow aims to automate this process.

The modular workflow of FEgrow allows us to experiment with functionalities, such as new optimisation or scoring methods. With the use of hybrid ML/MM structural optimisation, in particular, we aim to obtain reliable coordinates for the added R-groups. In this respect, the ANI neural network potential (within the ML/MM approximation) has already been shown to be capable of predicting protein–ligand binding poses in agreement with electron density distributions determined by X-ray crystallography^44^, and should be significantly more reliable than the general purpose force fields (such as UFF) that are typically used for structure refinement in de novo design packages. Updated machine learning potentials or semi-empiricial methods^65^ can readily be included in future versions of FEgrow.

Ligand designs are evaluated for simple molecular properties, and their binding affinity predicted using the gnina CNN scoring function. Despite the challenge of discriminating between relatively small functional group modifications, the scoring function performs quite well and is useful in providing initial guidance for a number of targets from the protein–ligand benchmark set used here. Nevertheless, we envisage the primary use of FEgrow being as a source of input structures for more rigorous free energy-based affinity predictions. We demonstrate this functionality here, using SOMD to calculate the relative binding free energies of 13 uracil-based inhibitors of the SARS-CoV-2 main protease. Using only a single crystal structure as input (PDB: 7L10) and the FEgrow workflow to build the remaining structures, we obtain excellent agreement with experimental binding affinities (MUE = 0.45 kcal/mol, *R*^2^ = 0.53).

We envisage future improvements including the use of a flexible receptor for the growth phase, and future use cases including seeding free energy calculations with multiple low energy conformers. The BACE(Hunt) target in Case Study I highlighted the difficulty of accurately including the energetics and effects on the binding affinity of displacing water networks in hydrated binding pockets. There does not currently appear to be a satisfactory means to include water networks into the optimisation or scoring phases of FEgrow, but output structures could be passed to molecular dynamics or Monte Carlo-based simulations to assess optimal hydration sites for predicted poses^66–68^. FEgrow is available for download from https://github.com/cole-group/FEgrow, and we welcome suggestions from the community for added functionality.

## Computational methods

### Free energy calculations

Structures of 13 inhibitors of the main protease (M^pro^) of SARS-CoV-2 were built using the FEgrow workflow and taken through to free energy calculations for accurate physics-based scoring. The PDB structure, 7L10, was used for the receptor. Missing residues (E47 and D48) were added using MODELLER^69^, which uses optimisation of a pseudo energy function for loop modelling, and hydrogen atoms were added using Chimera^20^, which includes options for optimisation of the hydrogen bond network. The BioSimSpace package^70^ was used for free energy setup, along with a relative binding free energy protocol used previously^71^. The lowest energy conformer for each ligand was parameterised with the GAFF2 force field, using the AM1-BCC charge model. The AMBER FF14SB^39^ force field was used for the protein, along with the TIP3P water model. Each ligand was then solvated in a 35 Å cube, or 90 Å cube in the presence of the protein. The bound and unbound structures then underwent a short equilibration using the default procedure in BioSimSpace^70^. Namely, the structure was minimised, then heated to 300 K in the NVT ensemble over a period of 10 ps. It was then equilibrated for a further 10 ps in the NpT ensemble at 300 K and 1 bar, using the Langevin thermostat and Berendsen barostat. Atoms in the protein backbone were restrained to their initial positions throughout, and a 8 Å nonbonded cutoff was applied.

The network of alchemical transformations was built manually to include cycle closures for error analysis, and is shown in Fig. S4. Table S6 shows that the absolute cycle closure errors are typically less than 0.5 kcal/mol, and less than 1 kcal/mol for all cycles. The overlap for each perturbation was determined using a maximum common substructure search to determine the atoms to be morphed. Each transformation leg was simulated using the SOMD software package^30^ for 4 ns, and the first 400 ps were discarded as equilibration. Eleven equally-spaced *λ* windows were employed between 0 and 1, along with the default soft core. The time step was set to 2 fs, with constraints applied to unperturbed hydrogen bonds. Simulations were performed in the NpT ensemble, using an Andersen thermostat with collision frequency of 10.0 ps^−1^ and a Monte Carlo barostat with a frequency of 25 time steps. Periodic boundary conditions and a tapered nonbonded cutoff distance of 10 Å were applied. Electrostatic interactions were calculated using the reaction-field method with a dielectric constant of 78.3 outside the nonbonded cutoff^72^. All transformations reported here were run in both forward and backward directions, and in duplicate. Free energy changes and their errors were calculated from the output with MBAR using the asymptotic covariance method^73^. Final free energies and their associated error bars (Fig. 5) were calculated from the network with the freenrgworkflows package^74^, using the method of Yang et al.^75^. All protocols used and raw data are provided in the accompanying Supporting Data (10.5281/zenodo.7112943).

## Supplementary information

Cole_PR File

Supplemental material

## Data Availability

Analysis of scaling factors used during geometry optimisation, background information on molecular property filters, full details of protein–ligand benchmark targets studied and CNN scoring function results, free energy networks and raw data for M^pro^ free energy calculations (Supplementary Information). Data accompanying this paper are freely available at 10.5281/zenodo.7112943.

## Code availability

FEgrow, and an accompanying tutorial, are freely available at https://github.com/cole-group/FEgrow. The version 1.0.2 of FEgrow used in this study is available for download from 10.5281/zenodo.7105647.
