# Supplementary material for: An open-source molecular builder and free energy preparation workflow
Source: Commun Chem. 2022 Oct 27;5:136. doi: 10.1038/s42004-022-00754-9 (PMC9607723; doi:10.1038/s42004-022-00754-9)
Supplement: Supplementary file 2 — Supplemental material [file 42004_2022_754_MOESM2_ESM.pdf]

Supplementary Information for: An Open-Source Molecular Builder and Free Energy Preparation Workflow

Mateusz K. Bieniek,[†] Ben Cree,[†] Rachael Pirie,[†] Joshua T. Horton,[†] Natalie J. Tatum,[‡] and Daniel J. Cole[*,†]

†*School of Natural and Environmental Sciences, Newcastle University, Newcastle upon Tyne NE1 7RU, United Kingdom*

‡*Newcastle University Centre for Cancer, Translational and Clinical Research Institute, Newcastle University, Newcastle upon Tyne NE2 4HH, United Kingdom*

E-mail: daniel.cole@ncl.ac.uk

Supplementary Note 1

Analysis of Lennard-Jones and Electrostatic Scaling Factors. As discussed in the section "Geometry Optimisation" in the main text, we follow the procedure recommended for the BOMB *de novo* design software[1] in scaling the Lennard-Jones radii and atomic charges when calculating the intermolecular energetics during geometry optimisation. This is to attempt to mitigate the rigid receptor approximation by i) allowing extra space in the binding pocket to accommodate ligand growth and ii) screening electrostatic interactions using an effective dielectric medium (ϵ).

Table S1 shows the effect of these choices of scaling factor on the correlation between gnina predicted binding free energies and experiment (R^2) for the set of thrombin inhibitors, and on the RMSD between the output R-group coordinates and crystal structure (see Case Study I in the main text for a full description). The correlation between gnina and experiment is very similar when using optimised structures either with no scaling, or with a scaling factor of 0.8 (and dielectric of 4). The correlation is lower using a scaling factor of 0.9, but on closer inspection this is due to a single outlier (Figure S1), and removing this point increases the correlation to 0.77.

It is interesting to note that, as shown in Figure S1, the predicted binding affinities become more favourable as the scaling factors tend towards one. This might be expected, as the van der Waals radii of the atoms approach their physical values, the structures are closer to the optimal binding poses as recognised by the CNN scoring function. This is corroborated by the observation that the agreement in structure between the FEgrow output and the known crystal structure (PDB: 2ZFF[2]) improves as the scaling factors are removed (RMSD decreases from 1.4 to 0.8 Å, Table S1). Despite these improvements in the absence of the scaling factors, we prefer to retain their use in the default behaviour of FEgrow to allow the possibility of more hits to be identified during prospective design. Nevertheless, the user is free to adjust them during run time.

Table S1: Effect of the LJ radii scaling factor and relative dielectric permittivity (ϵ) used during optimisation on the accuracy of predicted affinities and structures from FEgrow for the set of thrombin inhibitors (see Case Study I in the main text).

LJ scaling	ϵ	R^2	RMSD / Å
0.8	4	0.68	1.37
0.9	2	0.27	1.05
1.0	1	0.70	0.76

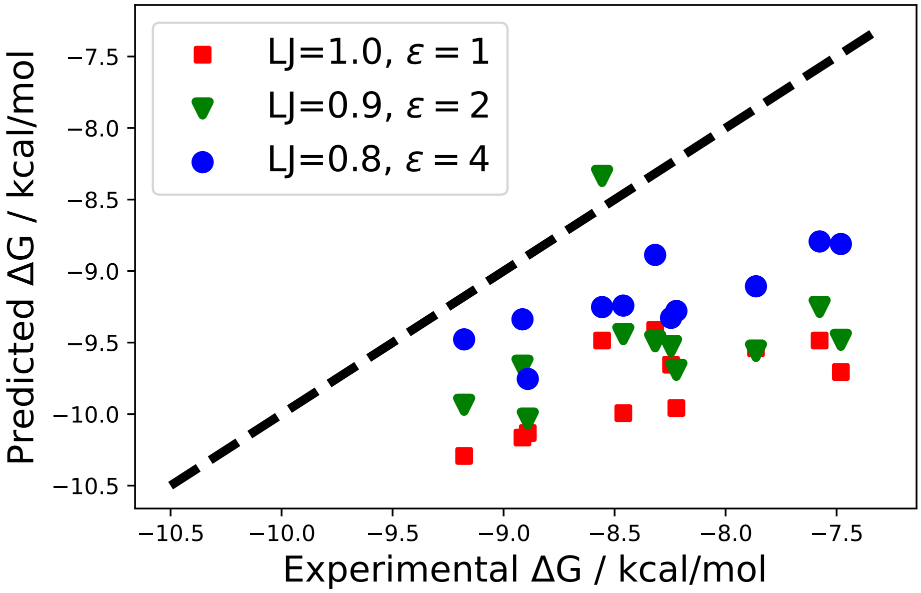

Figure S1: Effect of the LJ radii scaling factor and relative dielectric permittivity (ϵ) used during optimisation on the correlation between predicted and experimental binding free energies for the set of thrombin inhibitors (see Case Study I in the main text).

Supplementary Note 2

Molecular Property Filters. Here, we provide further information on the simple molecular property filters that are included in FEgrow.

Pan Assay Interference Compounds (or PAINS)[3] are molecules that bind non-specifically to multiple protein targets. This can lead to unwanted side effects and increased risk of toxicity, as well as often causing false positive results in high throughput screening. Common PAINS include toxoflavin, isothiazolones, hydroxyphenyl hydrazones, curcumin, phenolsulfonamides, rhodanines, enones, quinones, and catechols. It is worth noting that there are many instances of approved drugs containing PAINS, so they should be considered with caution.[4]

Brenk and coworkers proposed a list of unwanted substructures with undesirable pharmacokinetics or toxicity that they made use of in assembling screening libraries for neglected diseases.[5] This list of features includes sulfates and phosphates (likely resulting in unfavorable pharmacokinetic properties), nitro groups (mutagenic), 2-halopyridines and thiols (reactive).

The NIH filter (based on the work by Jadhav *et al.*[6] and Doveston *et al.*[7]) defines a list of unwanted functional groups. These are split into two groups: reactive functionalities and medicinal chemistry exclusions. The reactive functionalities include Michael acceptors, aldehydes, epoxides, alkyl halides, metals, 2-halo pyridines, phosphorus nitrogen bonds, α-chloroketones and β-lactams. The medicinal chemistry exclusions include groups such as oximes, crown ethers, hydrazines, flavanoids, polyphenols, primary halide sulfates and multiple nitro groups.

Finally, we include a synthetic accessability (SA) score.[8] This function returns a score of 1 for easy to synthesise and 10 for more challenging compounds. The score is based on a combination of fragment contributions derived through analysis of one million compounds from Pubchem, and a complexity penalty that accounts for the presence of large rings, non-standard ring fusions, stereocomplexity and size.

Table S2: PDB ID, number of R-groups grown, net ligand charge, and 2D common core structure for each target. Attachment vectors are labelled by "–R".

Target	PDB ID	Number of R-groups	Charge	Common Core
BACE	4DJW	16	+1	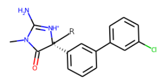
BACE(Hunt)	4JPC	31	+1	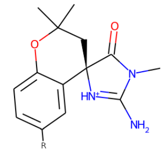
BACE(P2)	3IN4	12	+1	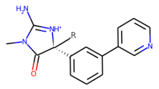
CDK2	1H1Q	16	0	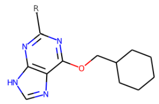
JNK1	2GMX	10	0	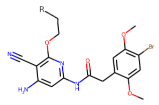
MCL1	4HW2	22	−1	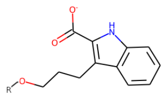
P38	3FLY	14	0	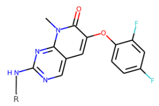

Table S3: (Continued) PDB ID, number of R-groups grown, net ligand charge, and 2D common core structure for each target. Attachment vectors are labelled by "–R".

Target	PDB ID	Number of R-groups	Charge	Common Core
PTP1B	2QBS	18	−2	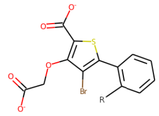
Thrombin	2ZFF	11	+1	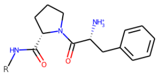
TYK2	4GIH	12	0	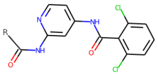

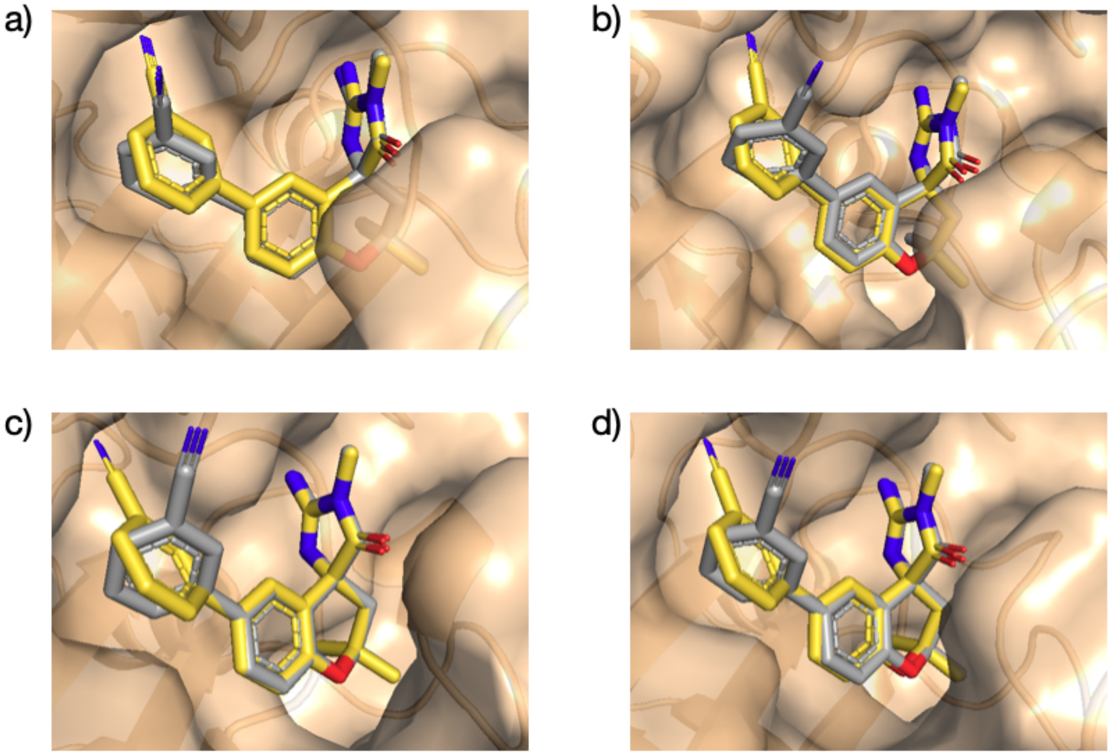

Figure S2: Overlay of protein–ligand benchmark dataset structures for the BACE(Hunt) target (PDB: 4JPC). Crystal structure in yellow and grown compound in grey. a) including water in the binding pocket as part of the receptor, b) using ANI for optimisation, c) using GAFF for optimisation, d) setting relative permittivity (ϵ) and the Lennard-Jones radii scaling factor to 1.0.

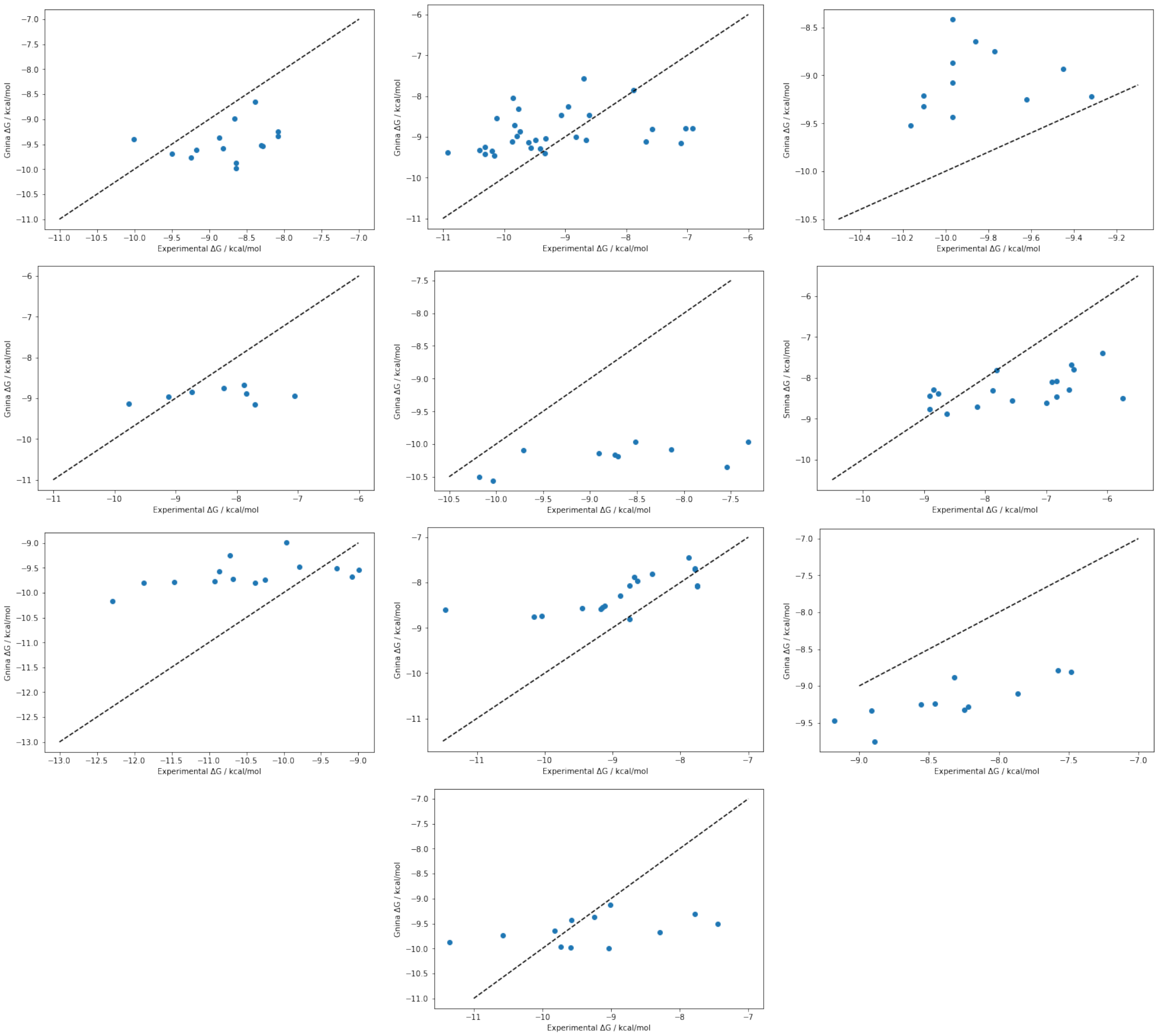

Figure S3: Absolute binding free energies of congeneric series of ligands taken from the protein-ligand benchmark set, using the gnina CNN affinity, compared with experiment. Protein targets from top left: BACE, BACE(Hunt), BACE(P2), CDK2, JNK1, MCL1, P38, PTP1B, Thrombin, TYK2.

Table S4: Root mean square error (RMSE) and correlation coefficient (R^2) between gnina CNN affinities (converted to free energies) and experimental binding free energy, calculated as $RT \times ln(IC_{50})$.

Target	RMSE / kcal/mol	R^2
BACE	0.94	0.00
BACE(Hunt)	1.23	0.03
BACE(P2)	0.89	0.00
CDK2	1.01	0.08
Jnk1	1.72	0.23
MCL1	1.19	0.27
P38	1.20	0.28
PTP1B	0.95	0.55
Thrombin	0.93	0.68
TYK2	1.03	0.20

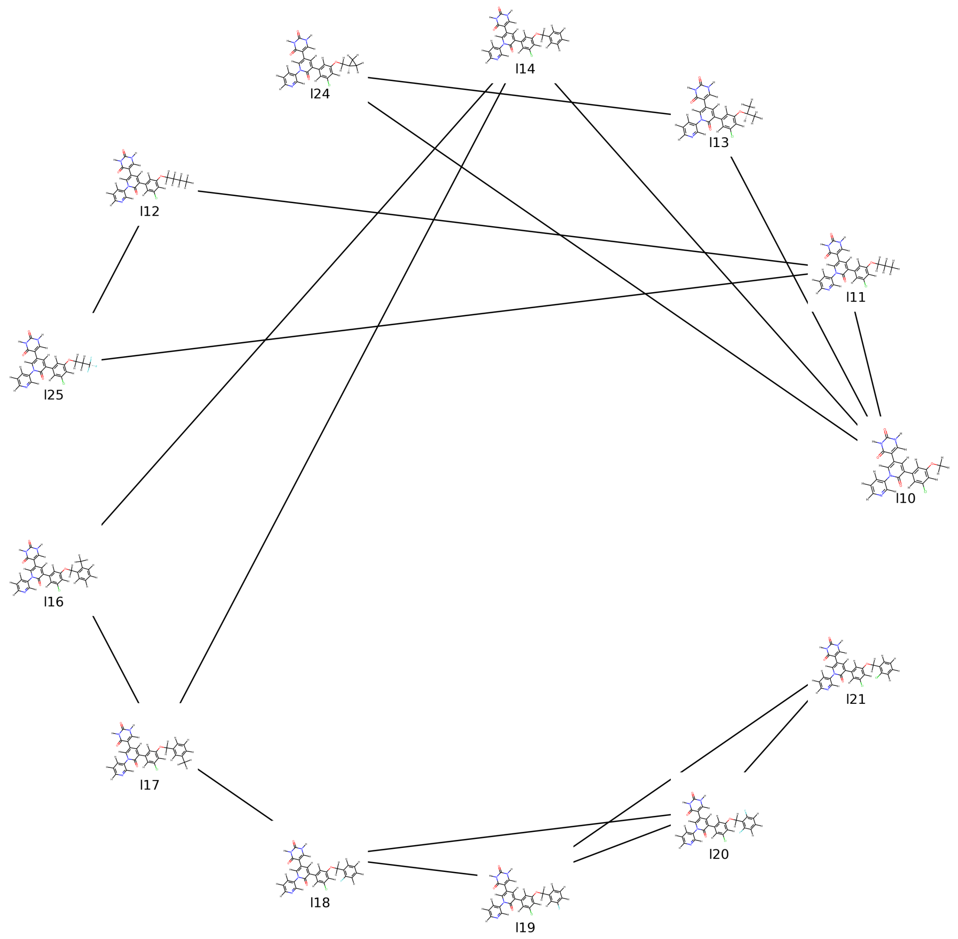

Figure S4: Network of alchemical transformations used for calculation of relative binding free energies of 13 analogs of the uracil-based M^{pro} inhibitors.

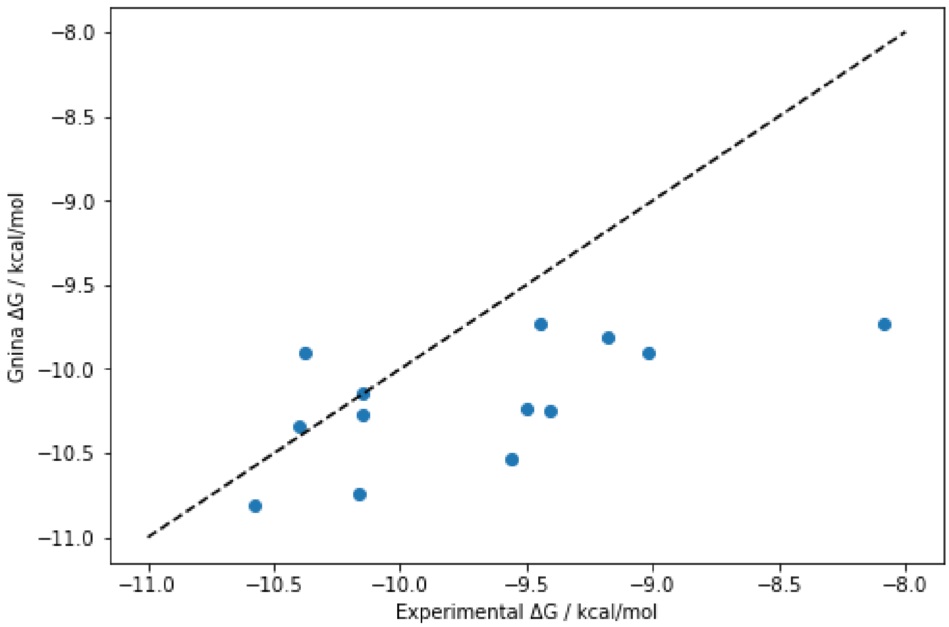

Figure S5: Comparison between gnina and experiment. Absolute binding free energies of 13 analogs of the uracil-based M^{pro} inhibitors using the gnina CNN affinity.

Table S5: Comparison between free energy calculations and experiment. Binding free energies of 13 analogs of the uracil-based M^{pro} inhibitors, relative to compound **10**.

Compound	ΔG(EXP) / kcal/mol	ΔG(SOMD) / kcal/mol
**10**	0	0
**11**	-1.37	-0.84
**12**	-0.94	-0.82
**13**	-1.10	-0.55
**14**	-1.33	-1.38
**16**	-1.48	-2.13
**17**	-1.42	-1.47
**18**	-2.33	-1.85
**19**	-2.07	-1.72
**20**	-2.09	-2.05
**21**	-2.5	-2.13
**24**	-2.07	-1.44
**25**	-2.31	-1.13

Table S6: Cycle closure errors for the network of M^{pro} inhibitors (Figure S3). Errors are calculated from the raw free energy data from SOMD, averaged over duplicate runs and forward/backward transitions.

Cycle	Cycle Closure Error (kcal/mol)
24–13–10	-0.98
14–16–17	-0.14
12–11–25	-0.46
18–20–19	0.01
19–20–21	0.43

Supplementary References

(1) Jorgensen, W. L. The Many Roles of Computation in Drug Discovery. *Science* **2004**, *303*, 1813–1818.

(2) Baum, B.; Mohamed, M.; Zayed, M.; Gerlach, C.; Heine, A.; Hangauer, D.; Klebe, G. More than a Simple Lipophilic Contact: A Detailed Thermodynamic Analysis of Nonbasic Residues in the S1 Pocket of Thrombin. *J. Mol. Biol.* **2009**, *390*, 56–69.

(3) Baell, J. B.; Holloway, G. A. New Substructure Filters for Removal of Pan Assay Interference Compounds (PAINS) from Screening Libraries and for Their Exclusion in Bioassays. *J. Med. Chem.* **2010**, *53*, 2719–2740.

(4) Capuzzi, S. J.; Muratov, E. N.; Tropsha, A. Phantom PAINS: Problems with the Utility of Alerts for Pan-Assay INterference CompoundS. *J. Chem. Inf. Model* **2017**, *57*, 417–427.

(5) Brenk, R.; Schipani, A.; James, D.; Krasowski, A.; Gilbert, I. H.; Frearson, J.; Wyatt, P. G. Lessons Learnt from Assembling Screening Libraries for Drug Discovery for Neglected Diseases. *ChemMedChem* **2008**, *3*, 435–444.

(6) Jadhav, A.; Ferreira, R. S.; Klumpp, C.; Mott, B. T.; Austin, C. P.; Inglese, J.; Thomas, C. J.; Maloney, D. J.; Shoichet, B. K.; Simeonov, A. Quantitative Analyses of Aggregation, Autofluorescence, and Reactivity Artifacts in a Screen for Inhibitors of a Thiol Protease. *J. Med. Chem.* **2010**, *53*, 37–51.

(7) Doveston, R. G.; Tosatti, P.; Dow, M.; Foley, D. J.; Li, H. Y.; Campbell, A. J.; House, D.; Churcher, I.; Marsden, S. P.; Nelson, A. A unified lead-oriented synthesis of over fifty molecular scaffolds. *Org. Biomol. Chem.* **2014**, *13*, 859–865.

(8) Ertl, P.; Schuffenhauer, A. Estimation of synthetic accessibility score of drug-like

molecules based on molecular complexity and fragment contributions. *J. Cheminformatics* **2009**, *1*, 8.
