# Supplementary material for: An open-source molecular builder and free energy preparation workflow
Source: Commun Chem. 2022 Oct 27;5:136. doi: 10.1038/s42004-022-00754-9 (PMC9607723; doi:10.1038/s42004-022-00754-9)
Supplement: Supplementary file 1 — Cole_PR File [file 42004_2022_754_MOESM1_ESM.pdf]

nature portfolio

# Peer Review File

## An Open-Source Molecular Builder and Free Energy Preparation Workflow

**Open Access** This file is licensed under a Creative Commons Attribution 4.0 International License, which permits use, sharing, adaptation, distribution and reproduction in any medium or format, as long as you give appropriate credit to the original author(s) and the source, provide a link to the Creative Commons license, and indicate if changes were made. In the cases where the authors are anonymous, such as is the case for the reports of anonymous peer reviewers, author attribution should be to 'Anonymous Referee' followed by a clear attribution to the source work. The images or other third party material in this file are included in the article's Creative Commons license, unless indicated otherwise in a credit line to the material. If material is not included in the article's Creative Commons license and your intended use is not permitted by statutory regulation or exceeds the permitted use, you will need to obtain permission directly from the copyright holder. To view a copy of this license, visit http://creativecommons.org/licenses/by/4.0/.

Reviewers' comments:

Reviewer #1 (Remarks to the Author):

The authors describe FEgrow, a software workflow for the structure preparation and energy pre-evaluation of protein ligand complexes aimed at automated relative binding free energy calculations. The software is a welcome contribution to the field and the manuscript is overall well written and presented.

I have a number of questions and concerns that should be addressed in a revised version of the paper.

First, it is mentioned that in the geometry optimisation phase, van der Waals radii are scaled to 0.8 and charges by 0.5. How was this choice calibrated/validated?

Concerning crystal water in BACE(hunt), the manuscript states: "Accurately including the energetics and effects on binding affinity of displacing water networks in hydrated binding pockets is evidently still a challenge." How is this accounted for in FEgrow? As this is a critical phase in the structure preparation task that FEgrow is designed for, an effort should be made to realistically model internal water molecules, for example in a retrospective study by matching known crstallographic water positions.

The manuscript states: "The network of alchemical transformations was built manually to include cycle closures for error analysis, and is shown in Figure S3." Which deviations from zero were obtained?

In the methods section it is not mentioned which charge model was used for GAFF2?

Also, a 10A cutoff was mentioned for the simulations. Is that a plain cut-off? For electrostatic interactions this is known to produce artifacts. Please comment.

Reviewer #2 (Remarks to the Author):

The manuscript of Bieniek et al. concerns the workflow (FEgrow) for building ligand poses in protein binding sites by growing functional groups from a core compound. The workflow is designed to set up input structures for relative binding free energy (RBFE) calculations. Since RBFE calculations based on molecular dynamics (MD) or Monte Carlo (MC) were shown to provide accurate predictions of protein-ligand binding affinity, their use in drug design projects is becoming increasingly common. Set-up of input structures for RBFE calculations is a crucial step because incorrectly prepared input structures can result in either failure of calculations or erroneous binding free energy predictions. Since manual preparation of input structures for a large number of RBFE calculations can be time-consuming, the automated set-up of RBFE calculation input is an important and relevant task. Therefore, I believe that the paper will be of interest for researchers dealing with computer-aided drug design. Since the FEgrow workflow is an open-source project and freely available, I believe that the paper will facilitate new advances in de novo drug design and protein-ligand binding free energy calculations.
The authors demonstrated the applicability and efficiency of their workflow by performing two case studies. In the first case study, FEgrow workflow was applied to 10 targets from the protein-ligand benchmark set to regrow congeneric ligands in the binding pockets. In the second case study FEgrow workflow was applied to generate input structures for RBFE calculations for a set of 13 congeneric inhibitors for the SARS-CoV-2 main protease. Average errors between binding free energies computed by FEgrow and experiment are acceptable for both case studies. RBFE calculations demonstrated sufficient correlation and low error with respect to experiment. The reported results provide convincing evidence of the applicability and efficiency of FEgrow workflow.

The manuscript is clearly written and well organized. The literature on de novo drug design and free energy calculations is summarized appropriately. The workflow design and obtained results are described consistently, coherently and with sufficient detail. I believe that the manuscript should be accepted with revisions.

The following are my comments and critique:
1. In the introduction, the authors mention several software packages capable of generating analogs of a hit compound by adding chemical functional groups to a rigid ligand core. These packages, therefore, have the same functionality as the workflow presented (FEgrow). The authors do not mention whether E-novo, Frag Explorer, DeepFrag, and Develop packages were used for set-up of input structures for RBFE calculations. If they can be used for this purpose, then it is unclear what are the advantages of FEgrow over these packages.

2. For SARS-CoV-2 main protease, RBFE of perampanel analogs were previously computed by Jorgensen research group using a workflow (BOMB/MCPRO) similar to FEgrow workflow. The authors compare their RBFE calculations with experiment but not with RBFE calculations reported by Jorgensen group. Since both workflows are based on the same approach for preparation of input ligand structures for free energy calculations, I believe the comparison of FEgrow/SOMD and BOMB/MCPRO would be appropriate. In particular, it is worth mentioning which workflow achieved better agreement between generated ligand structures with crystal structures and better accuracy of free energy calculations with respect to the experiment.

3. "Input and Constrained Conformer Generation" sections states that distance constraints to the common core are used for the energy minimization of generated conformers. The authors did not indicate which functon(e. g. root mean square distance) is used for these constraints.

4. According to "Geometry Optimisation" section, protein missing atoms, residues and hydrogen atoms are added using PDBFixer. Given that, it is unclear why the authors used Modeller and Chimera for the addition of missing residues and hydrogen atoms for SARS-CoV-2 main protease structure (PDB ID: 7L10) as indicated in the "Computational Methods" section.

5. In "Geometry Optimisation" section, paragraph 3 states: "The simulation uses the AMBER FF14SB force field …". I assume that "simulation" here applies to energy minimization mentioned in the previous sentence. The term "simulation" may be misleading: given that this is the first sentence of the paragraph, one can think that the simulation is a separate step following energy minimization. Therefore, I suggest either replacing "simulation" with "energy minimization" or edit this and previous sentence in such a way that the connection between these two terms is explicit.
6. "Case Study I: Protein-Ligand Benchmarks" section, paragraph 3: references to the Fig. 2 (a) and (b) for TYK2 and Thrombin systems are needed.

7. Table S1 in Supporting Information:  for each 2D structure of common core, I suggest highlighting atoms that were used as attachment points for growing functional groups (e. g. as it was done in figure 1 in the main text). This will make this figure more informative for the illustration of case study 1.

8. Both case studies: to quantitatively characterize overall agreement of ligand poses generated by FEgrow workflow with those presented in crystal structures, I suggest calculating root mean square deviation (RMSD) of functional groups of FEgrow generated ligand structures with respect to corresponding crystal structures. This quantity can be computed for each pair of structures (generated – crystal) and then averaged for all structures for the same protein target. Thus, one can estimate how accurate FEgrow can reproduce ligand poses in crystal structures in general and also compare performance of FEgrow for different protein targets.

9. Some details of free energy simulations are not presented neither in "Computational Methods"

section nor in Supporting Information. The missing details for the equilibration step are: simulation time, restraints applied, cutoff distance for nonbonded interactions, type of barostat and thermostat used. The missing details for the production step are: statistical ensemble, type of barostat and thermostat used. Also, the authors do not describe how the errors of free energies were calculated.

**Reviewer #1 (Remarks to the Author):**

The authors describe FEgrow, a software workflow for the structure preparation and energy pre-evaluation of protein ligand complexes aimed at automated relative binding free energy calculations. The software is a welcome contribution to the field and the manuscript is overall well written and presented.

I have a number of questions and concerns that should be addressed in a revised version of the paper.

**Response:** Thank you very much for your comments on the manuscript and helpful suggestions for improvement, which we address below.

*1) First, it is mentioned that in the geometry optimisation phase, van der Waals radii are scaled to 0.8 and charges by 0.5. How was this choice calibrated/validated?*

**Response:** This choice was made based on recommended settings in the BOMB *de novo* design software (which are unfortunately unpublished), and physical intuition. Since the optimisation stage is performed with a rigid binding site, it is desirable to include conformers that could be reasonably accommodated by some small adjustments in the receptor during subsequent MD simulations. Similarly, it is reasonable to include a factor to mimic the dielectric response that would be used to screen charges in the presence of a flexible binding site and solvent.

It is challenging to unequivocally validate this choice, but we have added a further analysis in the revised Supporting Information. Namely, for the thrombin target in Case Study I, we regenerate the structures of the R-groups and compute their gnina CNN binding affinities for a selection of Lennard-Jones scaling factors (0.8–1.0) and relative permittivities (1–4).

Interestingly, the correlation between predicted and experimental binding affinities is largely unchanged, but with no scaling the predicted affinities move to more negative values. The structure is also closer to experiment in the absence of scaling. We discuss these data further in the revised Section **S1**. We do prefer to keep the scaling behaviour as default in FEgrow, to allow for ligands to be more easily accommodated in the binding pocket, but also give users the option to change these values at run time.

*2) Concerning crystal water in BACE(hunt), the manuscript states: "Accurately including the energetics and effects on binding affinity of displacing water networks in hydrated binding pockets is evidently still a challenge." How is this accounted for in FEgrow? As this is a critical phase in the structure preparation task that FEgrow is designed for, an effort should be made to realistically model internal water molecules, for example in a retrospective study by matching known crystallographic water positions.*

**Response:** Crystal water molecules may be optionally retained in FEgrow, but only as part of the rigid receptor. That is, crystal water coordinates may be included in the input receptor structure by the user, and they will remain fixed during the geometry optimisation and scoring phases. We have expanded our description of this approach in the Geometry Optimisation section, in the revised manuscript.

Concerning the more general problem of how to include the effects of displaced water networks in pose generation and scoring, there is unfortunately no good solution (to the best of our knowledge). For example, one could imagine including extra water molecules as part of the ligand's flexible R-group, but how would one compare energetics of poses with differing numbers of water molecules? How many water molecules should be added to define the network? How would the scoring function account for this (water molecules are not included in the gnina CNN training data)?

In the revised manuscript, we have moved the discussion of crystallographic water to the Discussion section, where we also discuss possible future research directions, including for example the use of grand canonical Monte Carlo sampling for prediction of optimal water placement for binding poses generated by FEgrow.

*3) The manuscript states: "The network of alchemical transformations was built manually to include cycle closures for error analysis, and is shown in Figure S3." Which deviations from zero were obtained?*

**Response:** Cycle closure errors have been computed from the raw free energy data, and are now reported in **Table S6**. All cycle closure errors are < 1 kcal/mol.

*4) In the methods section it is not mentioned which charge model was used for GAFF2?*

**Response:** The AM1-BCC charge model was used, and this information has now been added to the revised Computational Methods.

*5) Also, a 10A cutoff was mentioned for the simulations. Is that a plain cut-off? For electrostatic interactions this is known to produce artifacts. Please comment.*

**Response:** Thank you for pointing out this oversight, we should have also mentioned that electrostatic interactions were computed using the reaction-field method with a tapered cut-off (at 10 Ang), so there is no abrupt cut-off. This is consistent with previous uses of SOMD (https://doi.org/10.1021/acs.jcim.0c00165). We have clarified the treatment of long-ranged interactions in the revised Computational Methods.

**Reviewer #2 (Remarks to the Author):**

The manuscript of Bieniek et al. concerns the workflow (FEgrow) for building ligand poses in protein binding sites by growing functional groups from a core compound. The workflow is designed to set up input structures for relative binding free energy (RBFE) calculations. Since RBFE calculations based on molecular dynamics (MD) or Monte Carlo (MC) were shown to provide accurate predictions of protein-ligand binding affinity, their use in drug design projects is becoming increasingly common. Set-up of input structures for RBFE calculations is a crucial step because incorrectly prepared input structures can result in either failure of calculations or erroneous binding free energy predictions. Since manual preparation of input structures for a large number of RBFE calculations can be time-consuming, the automated set-up of RBFE calculation input is an important and relevant task. Therefore, I believe that the paper will be of interest for researchers dealing with computer-aided drug design. Since the FEgrow workflow is an open-source project and freely available, I believe that the paper will facilitate new advances in de novo drug design and protein-ligand binding free energy calculations.

The authors demonstrated the applicability and efficiency of their workflow by performing two case studies. In the first case study, FEgrow workflow was applied to 10 targets from the protein-ligand benchmark set to regrow congeneric ligands in the binding pockets. In the second case study FEgrow workflow was applied to generate input structures for RBFE calculations for a set of 13 congeneric inhibitors for the SARS-CoV-2 main protease. Average errors between binding free energies computed by FEgrow and experiment are acceptable for both case studies. RBFE calculations demonstrated sufficient correlation and low error with respect to experiment. The reported results provide convincing evidence of the applicability and efficiency of FEgrow workflow.

The manuscript is clearly written and well organized. The literature on de novo drug design and free energy calculations is summarized appropriately. The workflow design and obtained results are described consistently, coherently and with sufficient detail. I believe that the manuscript should be accepted with revisions.

The following are my comments and critique:

**Response:** Thank you very much for your comments on the manuscript and helpful suggestions for improvement, which we address below.

*1) In the introduction, the authors mention several software packages capable of generating analogs of a hit compound by adding chemical functional groups to a rigid ligand core. These packages, therefore, have the same functionality as the workflow presented (FEgrow). The authors do not mention whether E-novo, Frag Explorer, DeepFrag, and Develop packages were used for set-up of input structures for RBFE calculations. If they can be used for this purpose, then it is unclear what are the advantages of FEgrow over these packages.*

**Response:** We are not aware of specific examples of outputs of these packages being used for free energy calculations, but the generative models in particular certainly could be used for this purpose. However, still today the majority of drug discovery campaigns use suggestions by experienced medicinal chemists to investigate structure-activity relationships around a lead series, rather than generative machine learning models.

Our motivation, therefore, with FEgrow was to replicate the success of the BOMB/MCPRO approach, which uses a molecular builder in conjunction with free energy calculations to help users to design extremely potent inhibitors of, for example, HIV reverse transcriptase. We have implemented these methods in an open-source, easy-to-use package that could be used by medicinal chemists to investigate their own designs. Essentially, we have automated the setup stages of free energy calculations that are currently performed manually.

As a result of our modular design of FEgrow, it was straightforward to then add the option of hybrid machine learning / molecular mechanics potentials for structural optimisation. Machine learning potentials show great promise and are significantly more reliable than, for example, the MM3 and UFF force fields that are used in Fragexplorer and DeepFrag, for example, for structural refinement. Again, these packages could be extended to add these functionalities, but we found it more convenient to write our own.

We have expanded the Discussion section of the revised manuscript to further discuss the philosophy and advantages of FEgrow.

*2) For SARS-CoV-2 main protease, RBFE of perampanel analogs were previously computed by Jorgensen research group using a workflow (BOMB/MCPRO) similar to FEgrow workflow. The authors compare their RBFE calculations with experiment but not with RBFE calculations reported by Jorgensen group. Since both workflows are based on the same approach for preparation of input ligand structures for free energy calculations, I believe the comparison of FEgrow/SOMD and BOMB/MCPRO would be appropriate. In particular, it is worth mentioning which workflow achieved better agreement between generated ligand structures with crystal structures and better accuracy of free energy calculations with respect to the experiment.*

**Response:** While the work by Jorgensen and co-workers is a tour de force in FEP-guided design (https://doi.org/10.1021/acscentsci.1c00039), the computational results tend to be presented and used only qualitatively, for example:

"FEP calculations were carried out and predicted gains in free energy of binding of 2−3 kcal/mol for methyl, fluorine, or chlorine substituents, which is large enough to usually yield observed benefits. The monomethyl analogues **16** and **17** were prepared and did show small improvement over **14** to 0.10 and 0.11 μM, respectively…"

Some quantitative FEP data is presented in Table 2 in the Jorgensen paper, but this is for the cyanophenyl series of inhibitors (we studied instead the uracil-based series). In any case, it is widely acknowledged that MCPRO strongly over-estimates the range of binding free energies: "Past experience with this FEP methodology has indicated that the range of the computed $\Delta\Delta G_b$ values is larger than observed by experiment…", due to methodological approximations

like use of a fixed protein backbone. The quantitative MCPRO data is thus unlikely to be competitive with our SOMD results.

Additionally, no (computational) structures or software are made available with the paper. Indeed, the difficulty of generating these workflows is our main motivation for developing the FEgrow open source package.

*3) "Input and Constrained Conformer Generation" sections states that distance constraints to the common core are used for the energy minimization of generated conformers. The authors did not indicate which function (e. g. root mean square distance) is used for these constraints.*

**Response:** Generated conformers are energy minimised using the UFF force field. Harmonic distance restraint functions in RDKit are applied to each atom in the core, with a very strong force constant of 10,000 kcal/mol/Ang^2, to effectively constrain the core to its initial position. This information has been added to the "Input and Constrained Conformer Generation" section of the revised manuscript.

*4) According to "Geometry Optimisation" section, protein missing atoms, residues and hydrogen atoms are added using PDBFixer. Given that, it is unclear why the authors used Modeller and Chimera for the addition of missing residues and hydrogen atoms for SARS-CoV-2 main protease structure (PDB ID: 7L10) as indicated in the "Computational Methods" section.*

**Response:** PDBFixer (https://github.com/openmm/pdbfixer) is a fast, convenient tool for preparing PDB structures for interactive work (e.g. in a Jupyter notebook), but does not give much control over the building process. On the other hand, MODELLER uses a well-validated algorithm for loop building based on optimisation of a pseudo energy function (including for example dihedral angle distributions in known structures) and Chimera includes consideration of hydrogen bond networks in assigning residue protonation states. These methods are more appropriate for setting up the more computationally intensive and rigorous free energy calculations. Further details on Chimera and MODELLER algorithms have been added to the revised Computational Methods.

*5) In "Geometry Optimisation" section, paragraph 3 states: "The simulation uses the AMBER FF14SB force field ...". I assume that "simulation" here applies to energy minimization mentioned in the previous sentence. The term "simulation" may be misleading: given that this is the first sentence of the paragraph, one can think that the simulation is a separate step following energy minimization. Therefore, I suggest either replacing "simulation" with "energy minimization" or edit this and previous sentence in such a way that the connection between these two terms is explicit.*

**Response:** Thank you, we have changed "simulation" to "energy minimisation" for clarity.

*6) "Case Study I: Protein-Ligand Benchmarks" section, paragraph 3: references to the Fig. 2 (a) and (b) for TYK2 and Thrombin systems are needed.*

**Response:** Thank you, we have now directed the reader to the appropriate figure in the revised manuscript.

*7) Table S1 in Supporting Information: for each 2D structure of common core, I suggest highlighting atoms that were used as attachment points for growing functional groups (e. g. as it was done in figure 1 in the main text). This will make this figure more informative for the illustration of case study 1.*

**Response:** Attachment points have now been labelled by "-R" in the revised Tables S2 and S3.

*8) Both case studies: to quantitatively characterize overall agreement of ligand poses generated by FEgrow workflow with those presented in crystal structures, I suggest calculating root mean square deviation (RMSD) of functional groups of FEgrow generated ligand structures with respect to corresponding crystal structures. This quantity can be computed for each pair of structures (generated – crystal) and then averaged for all structures for the same protein target. Thus, one can estimate how accurate FEgrow can reproduce ligand poses in crystal structures in general and also compare performance of FEgrow for different protein targets.*

**Response:** We have added the requested information to Figures 2 and 4 of the revised manuscript.

*9) Some details of free energy simulations are not presented neither in "Computational Methods" section nor in Supporting Information. The missing details for the equilibration step are: simulation time, restraints applied, cutoff distance for nonbonded interactions, type of barostat and thermostat used. The missing details for the production step are: statistical ensemble, type of barostat and thermostat used. Also, the authors do not describe how the errors of free energies were calculated.*

**Response:** The initial equilibration step uses the default procedure in BioSimSpace. (The structure is minimised, then heated to 300K in the NVT ensemble over a period of 10 ps. It is then equilibrated for a further 10ps at 300K and 1 bar, using the Langevin thermostat and Berendsen barostat. Atoms in the protein backbone are restrained to their initial positions throughout, and a 8 Ang nonbonded cutoff is applied.) Recall that a further 400ps of equilibration is employed with SOMD, as described in the manuscript.

The production stage uses the NpT statistical ensemble, with the Andersen thermostat and Monte Carlo barostat.

Errors in the free energies for a single run are calculated with MBAR using the asymptotic covariance method (https://doi.org/10.1063/1.2978177). The final error bars in Figure 5 are computed from the network with the freenrgworkflows package, using the method of Yang et al [https://doi.org/10.1002/jcc.26095].

These settings are all identical to the protocol employed previously (https://doi.org/10.1021/acs.jcim.1c00328), and we provide all input files and run scripts required in the Supporting Data. All information has now been added to the revised Computational Methods.

REVIEWERS' COMMENTS:

Reviewer #1 (Remarks to the Author):

The authors have satisfactorily addressed my comments.

Reviewer #2 (Remarks to the Author):

Agree with the revision, please accept
